# Computational Modeling of Synchrony in the Auditory Nerve in Response to Acoustic and Electric Stimulation

**DOI:** 10.3389/fncom.2022.889992

**Published:** 2022-06-17

**Authors:** Raymond L. Goldsworthy

**Affiliations:** Auditory Research Center, Caruso Department of Otolaryngology, Keck School of Medicine, University of Southern California, Los Angeles, CA, United States

**Keywords:** auditory neuroscience, cochlear implants, pitch perception, synchrony, auditory nerve

## Abstract

Cochlear implants are medical devices that provide hearing to nearly one million people around the world. Outcomes are impressive with most recipients learning to understand speech through this new way of hearing. Music perception and speech reception in noise, however, are notably poor. These aspects of hearing critically depend on sensitivity to pitch, whether the musical pitch of an instrument or the vocal pitch of speech. The present article examines cues for pitch perception in the auditory nerve based on computational models. Modeled neural synchrony for pure and complex tones is examined for three different electric stimulation strategies including Continuous Interleaved Sampling (CIS), High-Fidelity CIS (HDCIS), and Peak-Derived Timing (PDT). Computational modeling of current spread and neuronal response are used to predict neural activity to electric and acoustic stimulation. It is shown that CIS does not provide neural synchrony to the frequency of pure tones nor to the fundamental component of complex tones. The newer HDCIS and PDT strategies restore synchrony to both the frequency of pure tones and to the fundamental component of complex tones. Current spread reduces spatial specificity of excitation as well as the temporal fidelity of neural synchrony, but modeled neural excitation restores precision of these cues. Overall, modeled neural excitation to electric stimulation that incorporates temporal fine structure (*e.g*., HDCIS and PDT) indicates neural synchrony comparable to that provided by acoustic stimulation. Discussion considers the importance of stimulation rate and long-term rehabilitation to provide temporal cues for pitch perception.

## Introduction

Hearing is remarkable. People with the best sensitivity to musical pitch can discriminate tones that are less than a tenth of a percent apart. Those with the best sensitivity can hear sounds move in the environment by less than a degree. This precision requires perception of timing differences across ears on the order of tens of microseconds (Tillein et al., 2011; Brughera et al., 2013). Timing is essential to the auditory system. Special physiological mechanisms have evolved to encode timing of sounds with high fidelity (Joris et al., 2004; Joris and Yin, 2007; Golding and Oertel, 2012; Bahmer and Gupta, 2018). Auditory nerve fibers respond synchronously to incoming sounds for frequencies up to two thousand cycles per second, arguably with useful timing cues up to ten thousand cycles per second (Verschooten et al., 2019). Despite the many evolutionary developments required for precise coding of timing in the auditory system, and despite strong arguments for the importance of temporal fine structure for music and speech perception (Lorenzi et al., 2006), timing cues are largely discarded by sound processing for cochlear implants. This article describes results from computational modeling of cochlear implant stimulation followed by current spread and neuronal excitation. Results inform how neural synchrony is discarded by common stimulation strategies, and how synchrony can be restored by strategies that encode temporal fine structure of incoming sound into electrical stimulation.

Cochlear implants are remarkable. People who lose their hearing can hear again through an electrode array surgically implanted in their cochlea (Svirsky, 2017). Nearly a million people can hear because of cochlear implants. The quality of hearing is impressive with most recipients able to understand spoken speech in quiet without relying on lip-reading or other visual cues (Niparko et al., 2010). Music appreciation and speech reception in noise, however, tend to be poor (Looi et al., 2012). The fact that hearing with cochlear implants is not as good as normal is not surprising. Cochlear implants are limited in their ability to stimulate different regions of the auditory nerve (Middlebrooks and Snyder, 2007). The auditory nerve is comprised of ~30–40 thousand nerve fibers while cochlear implants use at most 22 stimulating electrodes (Loizou, 1999; Liberman et al., 2016). Furthermore, electrical current broadly spreads in the cochlea degrading the spatial specificity of stimulation (Landsberger et al., 2012).

This limit on spatial specificity is in sharp contrast to the capacity of cochlear implants to stimulate with temporal precision. Most cochlear implants control stimulation timing with microsecond precision (Shannon, 1992). This exquisite temporal precision, however, is poorly used by conventional sound processing. The most common sound processing for cochlear implants, Advanced Combinatorial Encoders (ACE), used on Cochlear Corporation devices, discards temporal fine structure in favor of encoding slowly varying envelope oscillations up to 300 cycles per second, even though there is clear evidence that timing information is important at least up to 2–4 thousand cycles per second (Dynes and Delgutte, 1992; Oertel et al., 2000; Joris et al., 2004; Verschooten et al., 2019).

There are historical reasons why ACE became commonly used today. Early sound processing for cochlear implants included simultaneous analog stimulation and strategies based on speech feature extraction (Loizou, 1999). Simultaneous analog stimulation caused interference between electrodes that was difficult to control and that produced unwanted fluctuations in loudness (Wilson and Dorman, 2008, 2018). Strategies built on speech feature extraction suffered from a different problem. Those strategies were limited by challenges of estimating speech features—fundamental and formant frequencies—in noisy environments. Consequently, the forerunner of the ACE strategy, known as Spectral Peak (SPEAK), broke through as a widely successful strategy based on amplitude modulation of pulsatile stimulation. By encoding spectral maxima directly, these stimulation strategies reliably conveyed essential speech information. This approach has consequently been referred to as speech processing rather than sound processing since it is quite effective for speech but less so for music.

Attempts have been made over recent decades to preserve this effective encoding of speech features provided by strategies like SPEAK and ACE while enhancing temporal information important for perception of pitch and melody in music. Peak-Derived Timing (PDT) triggers pulse timings based on the temporal fine structure of sound (van Hoesel and Tyler, 2003; van Hoesel, 2007; Vandali and van Hoesel, 2011). With PDT, sound is separated into overlapping frequency bands as done for ACE, but rather than discarding temporal fine structure, PDT triggers pulse timings based on local peaks in the filtered signal of each processing channel. A similar strategy was developed for MED-EL cochlear implants referred to as Fine Structure Processing (FSP), which schedules pulse timings on zero crossing rather than peaks, but the same principal applies. Further, while PDT applied the stimulus-derived timing for every electrode, FSP originally only applied the fine structure processing to the most apical electrode corresponding to bandpass filtering center frequencies up to 250 Hz (Riss et al., 2014). Variations of FSP have since been developed that use higher stimulation rates and present fine structure to the four most apical electrodes corresponding to bandpass filter center frequencies up to 950 Hz (FS4: Riss et al., 2014, 2016).

Results with such temporal encoding strategies have been mixed. Studies of PDT found little to no benefit on either pitch perception or spatial hearing (van Hoesel and Tyler, 2003; van Hoesel, 2007; Vandali and van Hoesel, 2011). Early reports for FSP found small but significant benefits on pitch without detriment to speech (Riss et al., 2014, 2016). More promising, recent studies suggest that benefits for pitch perception emerge from experience with the newly encoded information, with continued benefits after extended experience (Riss et al., 2016). There are many challenges to determining whether new strategies achieve the desired outcome of better encoding of temporal cues for pitch. Experience may be needed to fully learn to use these cues. Since long-term rehabilitation may be needed to learn how to use these cues, tools are needed to better characterize how well these cues are encoded for different stimulation strategies. Computational models of current spread and neural excitation can explicitly characterize cues available in the auditory nerve in response to cochlear implant stimulation. Careful characterization of these cues clarifies the extent that pitch perception of cochlear implant users is limited by the availability of these cues rather than by the brain's ability to learn to use them.

This article considers computational neural modeling of spatial and temporal cues in the auditory-nerve response for pitch perception. Cochlear implants can transmit spatial excitation cues with more deeply inserted electrodes evoking lower pitches (Shannon, 1983; Landsberger and Galvin, 2011). Cochlear implants also convey temporal cues with higher stimulation or modulation rates providing higher pitches (Tong et al., 1982; Zeng, 2002). There is considerable debate regarding optimal use of spatial and temporal cues for pitch perception for both acoustic and electric hearing (Eddington et al., 1978; Loeb, 2005; Cedolin and Delgutte, 2010; Kong and Carlyon, 2010; Oxenham et al., 2011; Miyazono and Moore, 2013; Marimuthu et al., 2016; Verschooten et al., 2019).

While it is unclear to what extent spatial and temporal cues contribute to pitch, the resulting resolution in normal hearing allows listeners to discriminate frequency differences <1% for a wide range of conditions (Moore et al., 2008; Micheyl et al., 2012). The best cochlear implant users despite having at most 22 stimulating electrodes, and despite discarding temporal fine structure, can distinguish pure tones based on frequency differences of about 1–5% from 500 to 2,000 Hz depending on frequency allocation of clinical processing (Goldsworthy et al., 2013; Goldsworthy, 2015). Pitch perception for complex tones, however, is comparably worse with cochlear implant users typically only able to discriminate differences in fundamental frequency of 5 to 20% in the ecologically essential range of 110–440 Hz (Goldsworthy et al., 2013; Goldsworthy, 2015). While it is difficult to characterize contributions of spatial and temporal cues for complex pitch (Carlyon and Deeks, 2002; Oxenham et al., 2004, 2011), work has clarified the extent that these cues are present and distributed in the auditory-nerve response (Cariani and Delgutte, 1996a; Plack and Oxenham, 2005).

For complex tones with fundamental frequencies in the range of human voices (100 to 300 Hz), cochlear implant filtering does not provide tonotopically resolved harmonic structure (Swanson et al., 2019). Consequently, implant users rely on temporal cues for discriminating complex tones in this range. Temporal cues for pitch become less effective with increasing rates with marked deterioration of resolution between 200 and 300 Hz. This leads many cochlear implant users to express frustration with melody recognition above middle C (~262 Hz) (Looi et al., 2012). It has been shown that discrimination improves for fundamental frequencies higher than 300 Hz, likely because of better access to place cues to make up for the impoverished rate cues (Goldsworthy, 2015; Swanson et al., 2019).

In the present article, the spatial and temporal cues associated with the frequency of pure tones and the fundamental frequency of complex tones is characterized in acoustic hearing using a computational model of the auditory periphery. This computational model has been rigorously validated on physiological recordings and behavioral measures (Zhang et al., 2001; Zilany et al., 2009, 2014). The same analyses are then performed to characterize the spatial and temporal cues available in cochlear implant stimulation followed by current spread and a point process model of neural excitation (Litvak et al., 2007; Goldwyn et al., 2012). Three cochlear implant stimulation strategies are examined including Continuous Interleaved Sampling (CIS), High-Definition CIS (HDCIS), and Peak-Derived Timing (PDT). These strategies were selected as representative of conventional stimulation that discards temporal fine structure (CIS) and newer strategies that actively encode acoustic temporal fine structure into electrical stimulation (HDCIS and PDT). Finally, an analysis of how stimulation rate affects neural synchrony is conducted to characterize information loss for devices that cannot stimulate with high pulse rates. The results inform the extent that acoustic temporal fine structure of incoming sound is transmitted into neural excitation.

## Materials and Methods

### Overview

Computational models were used to compare neural synchrony for acoustic and electric stimulation. Neural synchrony was quantified as vector strength. For acoustic stimulation, auditory-nerve fibers were simulated based on a computational model of the auditory periphery that has been validated with extensive physiological data (Zilany et al., 2014). For electric stimulation, cochlear implant stimulation was based on emulations of three stimulation strategies that differ in how temporal features of incoming sounds are encoded. The three strategies were Continuous Interleaved Sampling (CIS), High-Definition CIS (HDCIS), and Peak-Derived Timing (PDT). These strategies were probed using pure and complex tones. Output electrical stimulation patterns were processed through a model of current spread followed by a point process model of neuronal excitation (Litvak et al., 2007; Goldwyn et al., 2012).

### Stimuli

Stimuli were pure and complex tones generated in MATLAB® as 30 ms tones. Pure tones were generated as sinusoids in sine phase. Complex tones were generated by summing sinusoids in sine phase. Integer harmonic components were included from the fundamental to the highest harmonic <10,000 cycles per second (Hz). Harmonic components were summed with spectral attenuation of −6 dB per octave as typically occurs for complex sounds in nature (McDermott and Oxenham, 2008). All stimuli were calibrated to an input level of 65 dB sound pressure level (SPL) for the phenomenological auditory-nerve model and to have a peak value of one for cochlear implant processing.

### Computational Modeling of Auditory-Nerve Response to Acoustic and Electric Stimulation

#### Phenomenological Auditory-Nerve Model

Auditory-nerve activity was modeled using a phenomenological model of auditory processing that has been developed across multiple institutions (Zilany et al., 2014). This computational model includes aspects of the auditory periphery including a middle-ear filter, a feed-forward control-path, adaptive filtering to emulate cochlear mechanics, inner hair-cell transduction kinematics followed by a synapse model and discharge generator. Thus, it models multiple aspects of auditory physiology and has been validated with a wide range of physiological data as well as behavioral data from humans. While the model captures diverse aspects of the auditory periphery, it is referred to here as an auditory-nerve model since the focus is on the auditory-nerve response to acoustic stimuli. In this article, it will be referred to as the phenomenological auditory-nerve model to distinguish it from the point process neuron model used in cochlear implant simulations. The phenomenological auditory-nerve model was implemented with 256 fibers with logarithmically spaced characteristic frequencies from 125 to 8,000 Hz. The input level for all stimuli was specified as 65 dB SPL. The species parameter was set to human, which uses basilar-membrane tuning based on (Shera et al., 2002). The inner and outer hair-cell scaling factor was specified to model normal hearing. All three available fiber types (low, medium, and high levels of spontaneous discharge) were examined but significant differences were not observed related to neural synchrony between fiber types.

#### Cochlear Implant Processing

Cochlear implant stimulation was generated using emulations of CIS, HDCIS, and PDT. The first stage of processing for all emulations was to process stimuli through a bank of twenty-two filters with logarithmically spaced center frequencies from 125 to 8,000 Hz. Filters were second order with infinite impulse response with 3-dB crossover points geometrically positioned between center frequencies. For CIS, filter outputs were converted to temporal envelopes using Hilbert transforms. Temporal envelopes were then used to modulate 90 kHz pulsatile stimulation (~4,091 pulses per second per channel). For HDCIS, filter outputs were half-wave rectified and these rectified signals were used as high-definition temporal envelopes. These temporal envelopes were then used to modulate 90 kHz pulsatile stimulation. For PDT, a peak-detection algorithm was used to find local maxima in filtered outputs and a single pulse was scheduled for the corresponding electrode at that instance. All electrical pulses were modeled as biphasic with 25 μs phase durations and an 8-μs interphase gap.

#### Current Spread

A model was used to characterize the extent that current spread degrades temporal cues for pitch perception. It is well-known that the spatial cues for pitch are degraded by current spread, but it is poorly understood to what extent interactions of nearby electrodes leads to smearing of temporal cues. If nearby electrodes encode distinct temporal cues, and if current spreads from these electrodes to the same neural region, then the synchrony of neural response would be degraded. Current spread was modeled using an inverse law and assuming the electrode array was linear and parallel to modeled nerve fibers. Stimulation was designed for 22 electrodes with electrodes spaced 0.75 millimeters apart. The distance between electrodes to the closest neuron was 1 millimeter. The voltage at a neuron was calculated as the sum of 22 voltage sources modified based on the inverse law for voltage attenuation. Rationale for using simple models of electrode geometry and summation of electric fields have been described elsewhere (Litvak et al., 2007).

#### Neural Excitation

The modeled voltage source after current spread was used to drive a point process model of neuronal excitation as developed and described by Goldwyn et al. (2012). This point process model of individual neurons provides a compact and accurate description of neuronal responses to electric stimulation. The model consists of a cascade of linear and non-linear stages associated with biophysical mechanisms of neuronal dynamics. The details of the model are described in Goldwyn et al. (2012); but briefly, each processing stage is associated with biophysical mechanisms of neuronal dynamics. A semi-analytical procedure determines parameters based on statistics of single fiber responses to electrical stimulation, including threshold, relative spread, jitter, and chronaxie. Refractory and summation effects are accounted for that influence the responses of auditory nerve fibers to high-rate pulsatile stimulation. For the present study, the electrical current after current spread was normalized to an input level of 1 milliampere. For each neural location, neurons were modeled having thresholds from 0.1 to 0.8 milliamperes with increments of 0.05 milliamperes. All other model parameters for the point process model were as described by Goldwyn et al. (2012).

### Synchrony Quantified as Vector Strength

The study objective was to characterize synchrony to frequency and fundamental frequency in the auditory nerve for acoustic and electric stimulation. For modeled auditory-nerve response, computed using either the phenomenological or point-process models, vector strength was calculated based on spike timings (Goldberg and Brown, 1968; van Hemmen, 2013):


$$\begin{array}{l} VS_{AN}=|\frac{1}{N}\overset{N}{\underset{i=1}{\sum }}e^{j2\pi ft_{i}}| \end{array}$$

Where *N* is the number of action potentials, *f* is the frequency (or fundamental frequency) of interest, and *t*_*i*_ is the time of event. In the present study, a corresponding vector strength for cochlear implant stimulation was needed to compare synchrony at the level of the auditory nerve with synchrony in electrical stimulation patterns (both before and after current spread). Electrical stimulation is conveyed by cochlear implants with biphasic pulses with cathodic phase durations typically 25 μs in duration followed by symmetric anodic phases to balance charge. The total charge per phase was used to control the probability of neural spiking in the auditory nerve. As such, a modified version of vector strength was used to account for the probability of spiking based on charge delivered:


$$\begin{array}{l} VS_{CI}=\frac{\left|\sum_{i=1}^{N}q_{i}{\;*\;e}^{j2\pi ft_{i}}\right|}{\sum_{i=1}^{N}q_{i}} \end{array}$$

Where *q*_*i*_ is the normalized charge per phase of each biphasic pulse. This metric of vector strength falls between 0 and 1 and corresponds to the original definition but with events weighted by the probability of spiking approximated by the charge per phase delivered for each stimulating pulse.

### Analysis of Interspike Intervals

As has been described in the literature, electric stimulation produces a high degree of synchronous firing of auditory-nerve fibers (Hong and Rubinstein, 2006; Hughes et al., 2013). Pulse trains with abrupt stimulus onsets can produce hyper-synchronization to the first pulse followed by refractoriness and potentially hyper-synchronization not to the next pulse in a sequence but to alternating pulses in the sequence. This putative response to pulse trains with abrupt onsets has been shown in computational models as well as measures of the electrically evoked compound action potential. Hyper-synchronization to alternating pulses would not be detected by vector strength since only the relative phase of the stimulus cycle is considered. Consequently, interspike intervals were analyzed. Interspike interval distributions were calculated based on 100 iterations of a 500 Hz input tone that was 100 ms in duration. Current level per pulse was chosen so that the stimuli evoked ~100 spikes per second.

### Vector Strength Across Frequencies and Across Fundamental Frequencies

Analyses were conducted to compare vector strength of modeled auditory-nerve activity for acoustic and electric stimulation for pure tones for semitone-spaced frequencies from 125 to 8,000 Hz and for complex tones for semitone-spaced fundamental frequencies from 55 to 1,760 Hz (corresponding to musical notes from A1 to A6). Response properties were characterized for a representative frequency or fundamental frequency, and vector strength was calculated for all frequencies and fundamental frequencies. Interpretation of results focus on information present in electric stimulation and how synchrony is affected by current spread and neural response.

### Effect of Stimulation Rate on Neural Response

The analyses described in the previous sections were conducted with modeled stimulation rate of 90,000 pulses per second (pps), which is possible with state-of-the-art cochlear implants. However, the most common implants in use today only support stimulation rates of 14,400 pps, which must be distributed across electrodes. Typically, electrode selection is used to distribute stimulation across 6–8 electrodes per stimulation cycle dividing to 1,800 pps per electrode. Further, there are many legacy implants that only support slower stimulation. The N22 implant from Cochlear Corporation can only stimulate at ~3,500 pps (the exact rate depends on the stimulus configuration). Even with an aggressive selection of only five electrodes per stimulation cycle, the resulting electrode stimulation rate would only be 700 pps per electrode. To conclude this article, the effects of reduced stimulation rate on modeled neural response is examined.

Front-end spectral filtering was implemented using the same bank of 22 band-pass filters described in previous sections. Temporal envelopes including temporal fine structure were derived as used for HDCIS, but channel selection was implemented using an emulation of Advanced Combinatorial Encoders (ACE). This combination of high-definition temporal envelopes with electrode selection is referred to here as high-definition ACE (HD-ACE). Channel selection was specified to be increasingly aggressive to counteract decreases in stimulation rate. Eight active electrodes were used for the 90,000 pps stimulation rate, six for the 14,400 pps stimulation rate, and four for the 3,500 pps stimulation rate. Current spread was modeled as in previous sections. Neural response was modeled for neurons having fiber locations from 0 to 30 millimeters in 0.1-millimeter increments with the electrode array parallel and 1 millimeter away. The most basal electrode was modeled as perpendicular to the 0-millimeter neural location. As in previous sections, neuron thresholds between 0.1 and 0.8 millivolts in 0.1-millivolt increments were modeled.

## Results

### Modeled Neural Response to Acoustic Stimulation

**Figure 1:**
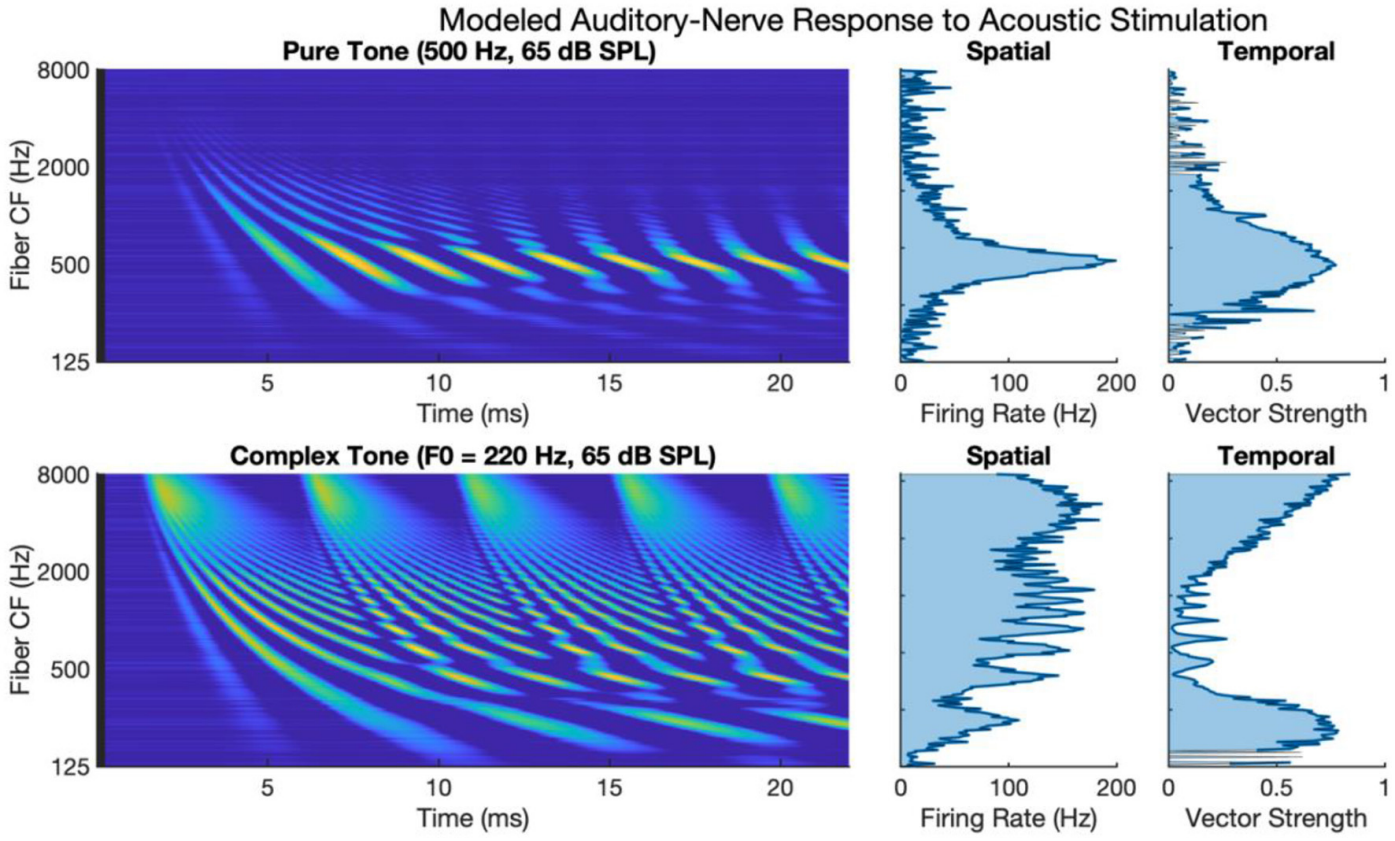
Modeled auditory-nerve response to acoustic stimulation for pure and complex tones. Left panels show spectrogram representations of average fiber firing rate for 256 fibers logarithmically spaced between 125 and 8,000 Hz. Middle panels show spatial excitation cues as firing rate averaged across time. Right panels show temporal cues as vector strength to the input tone frequency of fundamental frequency.

Figure 1 shows auditory-nerve activity when driven by acoustic stimulation as modeled by the phenomenological model of auditory processing (Zilany et al., 2014). The upper subpanels of Figure 1 show auditory-nerve activity and response metrics for a 500 Hz pure tone. It has been shown in the literature that the auditory nerve responds with well-defined tonotopy and with synchronous phase-locked spiking to acoustic frequency for frequencies at least as high as 2 to 4 kHz (Dynes and Delgutte, 1992), though noting arguments and evidence for phase-locked firing as high as 10 kHz (Verschooten et al., 2019). The modeled activity accounts for tonotopy and synchrony with a clearly defined tonotopic response centered on the characteristic frequency of the tone. The response exhibits a high degree of synchronous excitation periodically every 2 ms. The periodic excitation travels from base to apex capturing the established behavior of the cochlear traveling wave. The calculated metric of synchrony shows that vector strength to the 500 Hz frequency is high with a maximum of 0.88.

The lower subpanels of Figure 1 show modeled auditory-nerve activity and response metrics for a complex tone with a fundamental frequency of 220 Hz. In terms of spatial excitation cues, there are well-defined spatially resolved peaks in the excitation pattern for the fundamental and roughly for the first 8–10 harmonics. Above the tenth harmonic, the excitation peaks are not well-defined in terms of tonotopic response. Importantly, the synchrony of the response is also well-defined with spatially resolved peaks in the patterning of vector strength. Synchrony near the fundamental is high reaching a maximum of 0.90 near the fundamental. At that point, synchrony fluctuates in between harmonic components. For fibers having a characteristic frequency near a harmonic, the fiber response is not synchronous to the fundamental; instead, response phase-locks to the temporal fine structure of the dominant harmonic (Delgutte and Kiang, 1984; Sachs et al., 2002; Kumaresan et al., 2013). In between harmonic components, two or more components interact to produce strong oscillations at the fundamental frequency. This alternating pattern of synchrony to the fundamental has been referred to as a fluctuation profile and has been hypothesized as important for pitch (Carney et al., 2015; Carney, 2018). For fibers with characteristic frequencies above 2 kHz, as filters broaden allowing more harmonic components to interact, the periodicity of the fundamental dominates the temporal response, and the vector strength increases with a maximum of 0.97 in this region.

### Modeled Neural Response to Electric Stimulation

**Figure 2:**
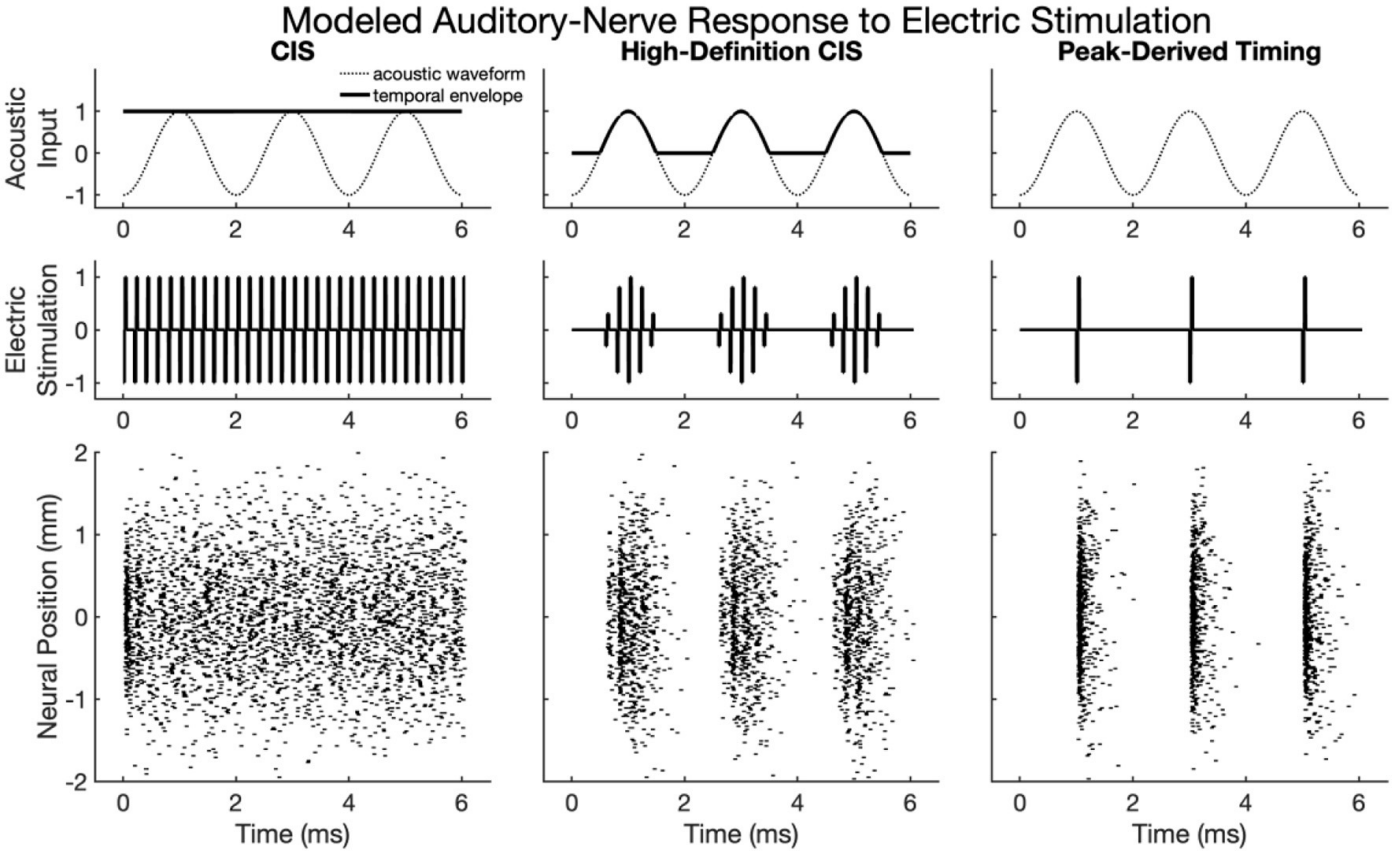
Modeled auditory-nerve response to electric stimulation for three cycles of a 500 Hz pure tone as encoded by three different cochlear implant stimulation strategies. For CIS, a relatively slow envelope extraction procedure discards incoming temporal fine structure, and the derived envelope is used to modulate constant-rate pulsatile stimulation. For HDCIS, the envelope is “high-definition” in that it is a rectified version of the filtered input signal and thus retains the temporal fine structure. For PDT, individual electric pulses are triggered based on the temporal maxima of the filtered input signal. The lower subpanels show modeled neural response to stimulation with the neural position indicating the relative position of the modeled neural elements with a value of 0 millimeters indicating a neuron closest to the electrode array, which is modeled as parallel to the nerve fiber and 1 millimeter away.

Figure 2 shows modeled neural activity for three cycles of a 500 Hz pure tone for the CIS, HDCIS, and PDT stimulation strategies implemented with a stimulating pulse rate of 90,000 pps. This figure clarifies how different stimulation strategies encode temporal cues of pure tones into electric stimulation and how neural excitation is predicted to respond. For this figure, only a single electrode was modeled for stimulation. The driving stimulus is represented in the upper subpanels as a sinusoid. For CIS, a relatively slow temporal envelope is derived using a Hilbert transform then used to modulate constant-rate pulsatile stimulation. Importantly, the pulse rate used for CIS is independent of the incoming tonal frequency and thus does not convey temporal cues for pitch associated with the incoming sound. For HDCIS, the envelope that is derived and used to modulate pulsatile stimulation is a half-wave rectified version of the filtered acoustic waveform, which does contain temporal fine structure associated with the frequency of the incoming sound. The electric stimulation represented in the middle subpanels clearly indicates the encoding of this cycle-by-cycle amplitude modulation of pulsatile stimulation. The PDT strategy does not derive envelopes but instead schedules pulsatile activity based on peak detection implemented on the filtered acoustic waveform. This approach is like the fine structure processing used on MED-EL devices, but the latter based on zero crossings rather than peak detection. In either case, the electric stimulation clearly conveys the periodicity of the incoming acoustic frequency as a singular electric pulse for each cycle.

The lower panels of Figure 2 show the modeled neural response for neurons with thresholds having a range of 0.1–0.8 milliamperes (with 0.1 milliampere increments) for 201 fiber locations. The fiber locations, or neural positions, are given relative to a location of 0 millimeters corresponding to the closest neuron to the stimulating electrode, but with the stimulating electrode 1 millimeter away from that closest neuron. With this range of thresholds and neural locations, the modeled neural activity shows a range of responses. For CIS, the stochastic nature of the point process model, coupled with the range of modeled thresholds, causes spike timings to quickly desynchronize after having a strong onset response. For HDCIS, the range of modeled thresholds and spatial locations provides a clear synchrony of spiking both the pulse rate and acoustic periodicity but noting that no modeled neurons responded to the last stimulating pulse of the stimulus. For PDT, the precise temporal pattern of the electric stimulus produces synchronous behavior in the modeled neural elements.

### Interspike Intervals in Response to Electric Stimulation

**Figure 3:**
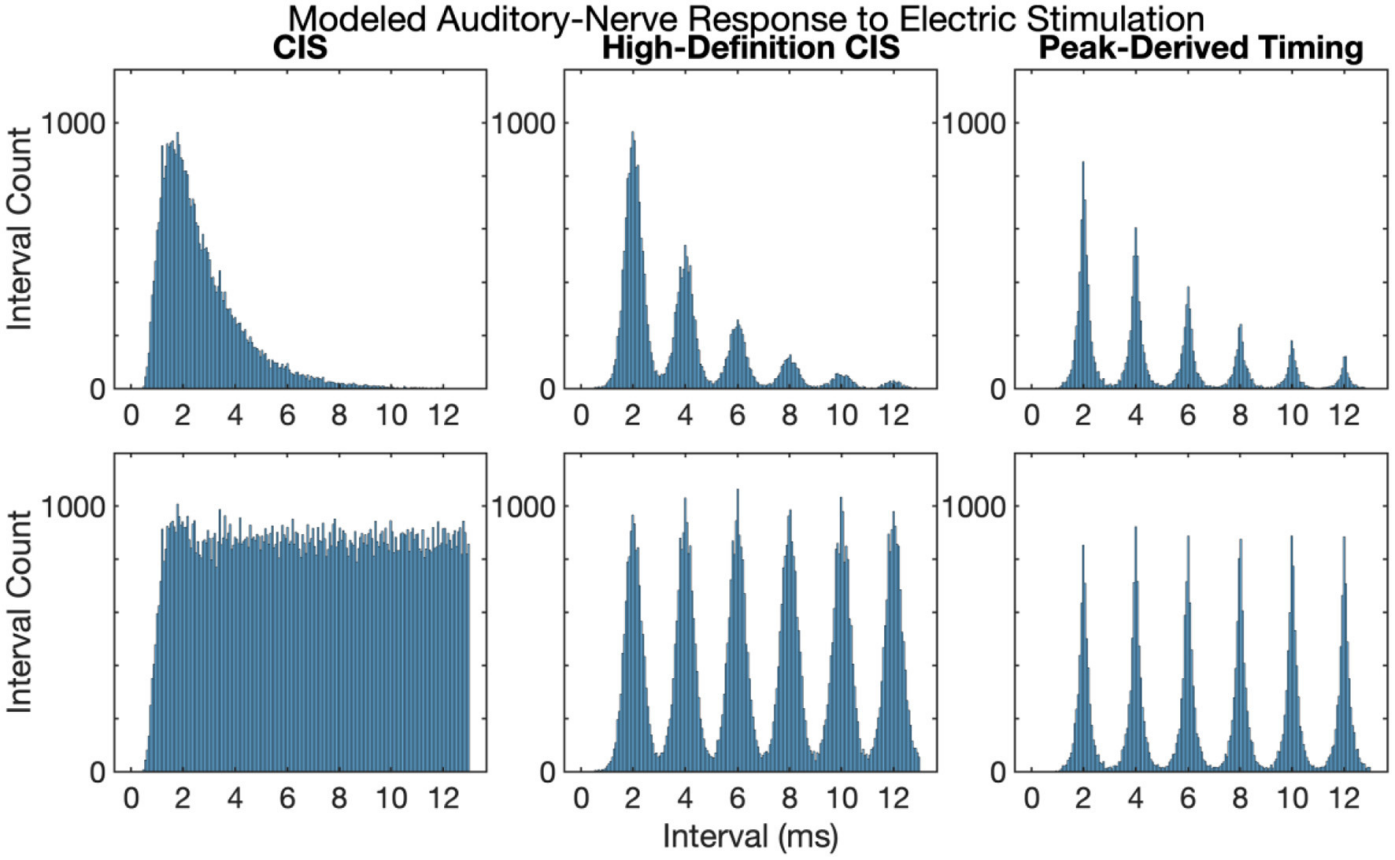
First and all-order interspike intervals for modeled auditory-nerve responses to electric stimulation depicted in Figure 2. Interspike interval distributions estimated from 10,000 interspike intervals with the pulse rates as indicated. Current level per pulse was chosen so that the stimuli evoked ~100 spikes per second.

Figure 3 shows first and all-order interspike intervals for modeled auditory-nerve responses described in the previous section. The CIS strategy produces a high-rate pulse train, the rate of which is independent of the frequency of the acoustic input, the neural response does not have interspike intervals representative of the 500 Hz periodicity of the input acoustic tone. Instead, the CIS strategy produces interspike interval distributions qualitatively like high-rate distributions recorded from cat auditory-nerve fibers (Miller et al., 2008) and as previously modeled by Goldwyn et al. (2012). In contrast, both HDCIS and PDT produced interspike intervals with clear histogram periodicities at the desired 2 ms periodicity of the input acoustic tonal frequency. The roll-off of histogram interval counts for the first-order statistics and flattened interval counts for the all-order interval counts agrees with the recordings made in cat auditory nerve fibers by Mckinney and Delgutte (1999). These modeling results suggest that HDCIS and PDT convey temporal cues for pitch beyond what is characterized by vector strength. These results are important because cochlear implant stimulation can sometimes introduce unwanted timing distribution of neural events. Specifically, abrupt pulsatile stimulation may lead to hyper-synchronization to an initial pulse with recovery and synchrony to odd pulses. Such artifactual response properties would not diminish vector strength but would affect interspike intervals. The results here suggest that such artifactual hyper-synchronization to alternating stimulation cycles is not occurring and that the interspike intervals produced by HDCIS and FSP strategies is comparable to that observed with acoustic stimulation (Mckinney and Delgutte, 1999).

### Comparison of Cochlear Implant Stimulation Strategies for Pure Tones

**Figure 4:**
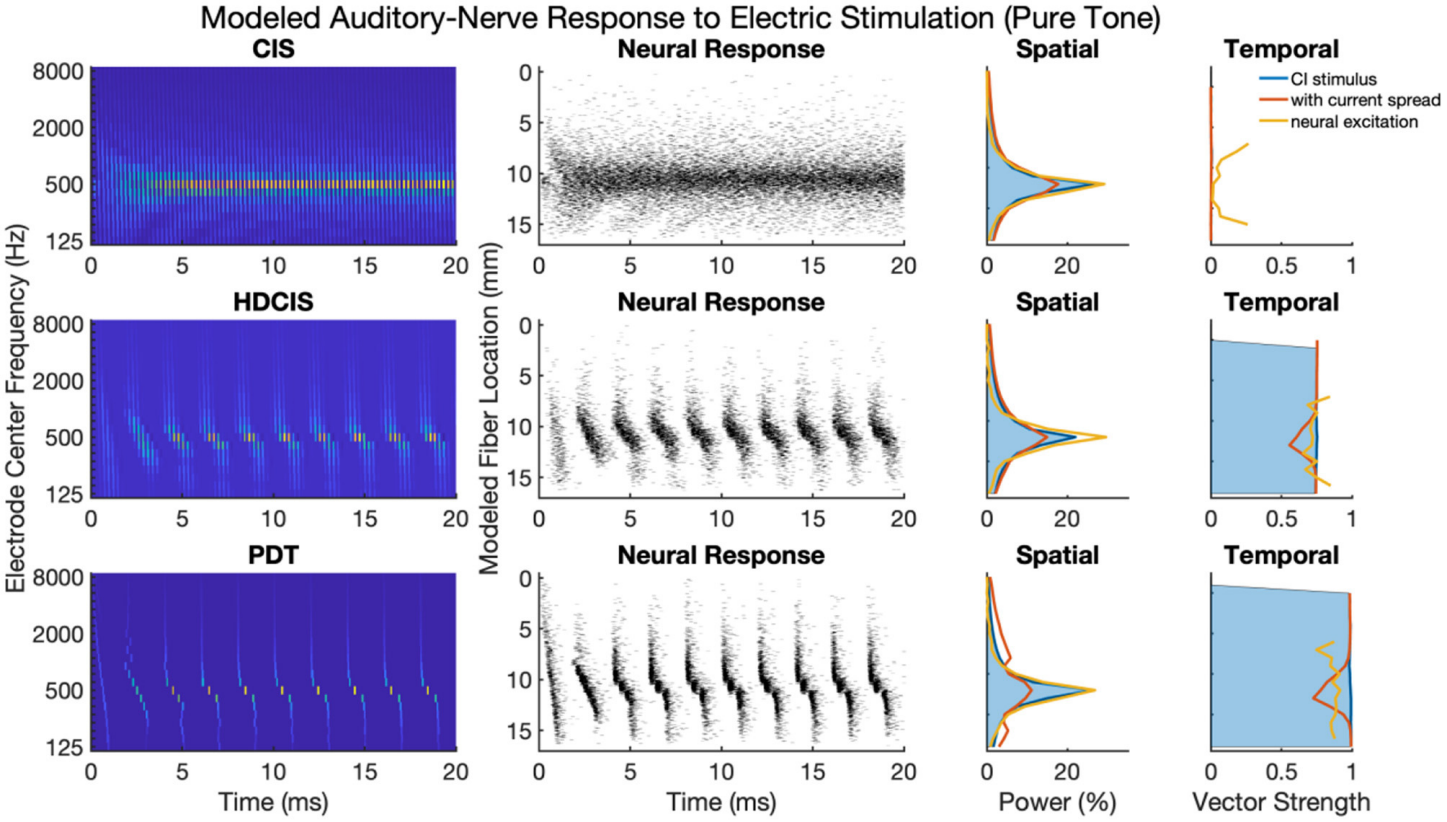
Cochlear implant stimulation, neural response, and derived metrics of spatial and temporal cues for a 500 Hz pure tone. For the electrodogram representations of the left panels, the electric potential was normalized to a peak of 1 millivolt as indicated by the color bar and only the anodic phase of stimulation is depicted. The neural responses were calculated for neuron thresholds from 0.1 to 0.8 in 0.1 microamperes steps and the current level of the electrical stimulus after current spread was specified to produce ~100 spikes per second.

Figure 4 shows cochlear implant stimulation and derived metrics for a representative 500 Hz pure tone. The tonotopic aspect of the stimulus is well-defined with the strongest response seen in the filter channel with center frequency of 500 Hz. The encoding of the acoustic temporal fine structure of the tone—the cycle-by-cycle periodicity—is strikingly different with CIS compared to HDCIS and PDT. Specifically, CIS discards acoustic temporal fine structure and stimulates using constant-rate pulsatile stimulation, with the pulse rate independent of the incoming sound. Consequently, synchrony quantified as vector strength between the stimulation pattern and the tone frequency is near zero. This result is not surprising since it is known that CIS discards acoustic temporal fine structure and only encodes envelope modulations (Svirsky, 2017). In contrast, HDCIS and PDT have markedly improved synchrony to the input tone frequency, HDCIS has a vector strength of 0.79 at the maximally excited filter, while PDT has a vector strength of 0.98 at its maximally excited filter.

The derived metrics of spatial and temporal cues illustrate the effects of modeled current spread and neural excitation. Current spread, naturally, spreads the spatial response reducing the relative power of the maximally excited filter. Interestingly, the compressive nature of modeled neural excitation increases the relative power of the maximally excited filter. This effect is likely caused by low-level filters not producing sufficient electrical stimulation to reach threshold of modeled neurons. The synchrony of response is also affected by modeled current spread and neural excitation. Since cochlear implant sound processing used here incorporates recursive filtering with phase-delay characteristics comparable to traveling wave mechanics of the cochlea, the energy profile for each cycle peaks first in high-frequency filters with notable elongation near the peak resonant filter (*i.e.*, the spectral maximum). This temporal spreading of fine structure results in peak energy occurring first in high-frequency channels with maximal temporal spreading near the filter with highest spectral output. Consequently, current spread results in temporal fine structure of one filtering channel to diminish the temporal precision of adjacent channels. This is observed in the marked reduction of synchrony following current spread. However, for HDCIS and PDT, vector strength is higher after modeling the neural response using the point process model with variable thresholds. This recovery of synchrony is likely driven by the synchronization of modeled spiking to charge accumulation on a cycle-by-cycle basis with rest sufficient period in between periods of the encoded temporal fine structure.

### Comparison of Cochlear Implant Stimulation Strategies for Complex Tones

**Figure 5:**
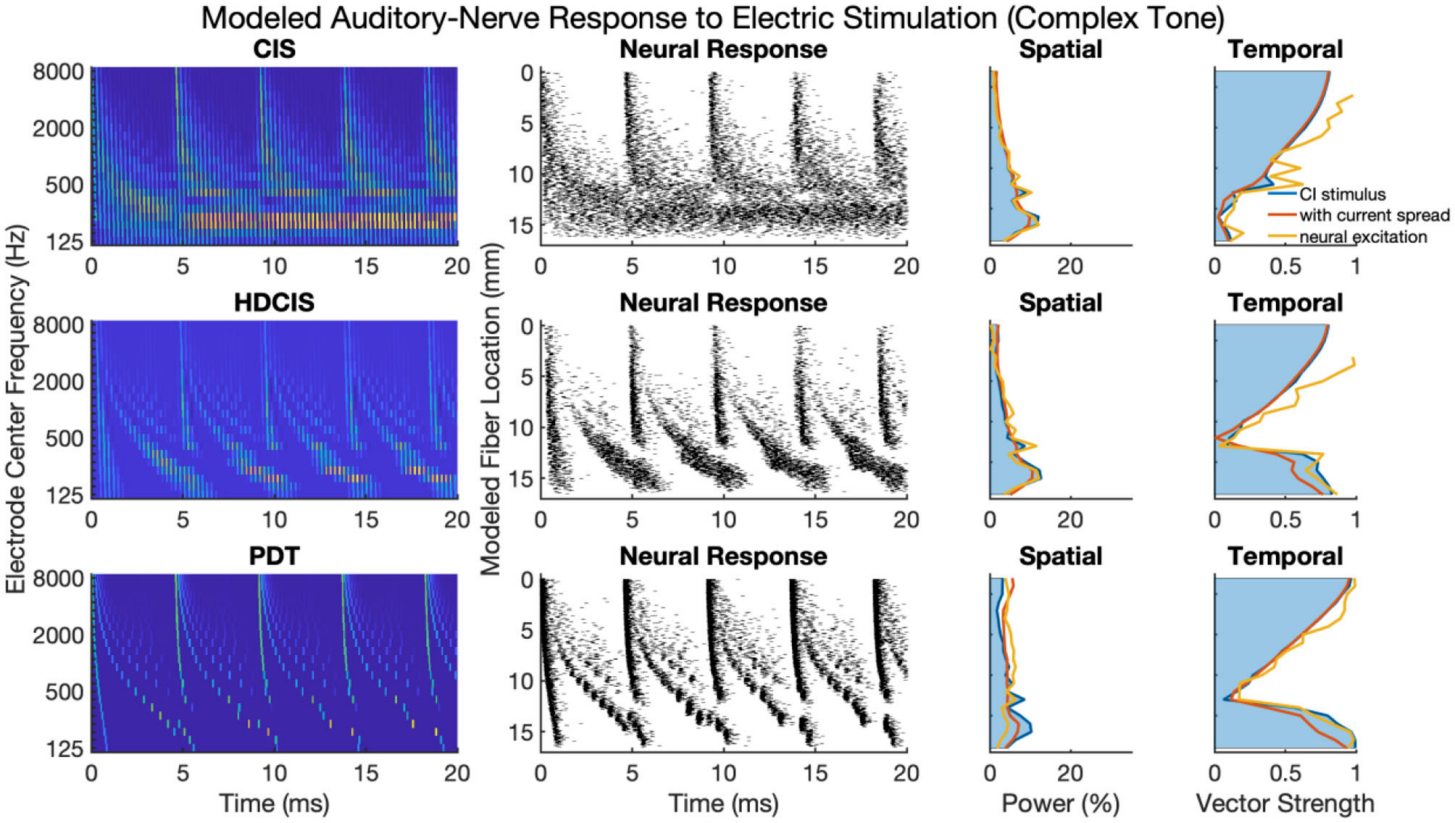
As Figure 4 but for complex tones with fundamental frequency of 220 Hz.

Figure 5 presents a representative analysis of the spatial and temporal cues associated with complex tones. For this representative analysis, a harmonic complex with fundamental frequency of 220 Hz is processed through the three stimulation strategies. The spatial response to the harmonic tone is nearly identical for the three strategies, with the response quantified as the proportion of charge delivered to each electrode. Note that proportional charge is normalized here, clinical implementation of PDT will likely require higher charge on low-frequency channels since lower pulse rates require more charge per pulse to obtain audibility thresholds and comfortable listening levels (Goldsworthy and Shannon, 2014; Bissmeyer et al., 2020; Goldsworthy et al., 2021, 2022). For all three stimulation strategies, the fundamental frequency clearly produces a resolved peak in the spatial pattern, which occurs in the stimulation pattern as well as after modeling current spread and neural excitation. Compared to the spatial cues available in acoustic hearing (see Figure 1), the spatial cues available in electric hearing are poorly represented beyond the fundamental frequency. The modeled neural response to acoustic input of Figure 1 depicts 8–10 harmonics that produce spatially resolved excitation, which is in general agreement with the physiological and psychophysical literature (Plack and Oxenham, 2005). In contrast, with only 22 logarithmically spaced filters, only the fundamental and first harmonic produced spatially resolved harmonics, the latter being substantially degraded by current spread but noting that the modeled neural response enhances the response.

The temporal cues associated with the fundamental frequency of complex tones strikingly differ for CIS compared to HDCIS and PDT. For all three stimulation strategies, temporal cues quantified as vector strength of synchrony between the electric stimulus and modeled neural excitation with the incoming acoustic stimulus is particularly high for high-frequency spectral regions where unresolved harmonics dominate and produce strong temporal periodicity. However, the stimulation strategies differ in the temporal response associated with the fundamental. Specifically, temporal cues associated with the fundamental frequency of the stimulus are not available in the low-frequency spectral region for the CIS strategy but are clearly present and markedly high for both HDCIS and PDT.

### Synchrony Across Frequencies for Pure and Complex Tones

**Figure 6:**
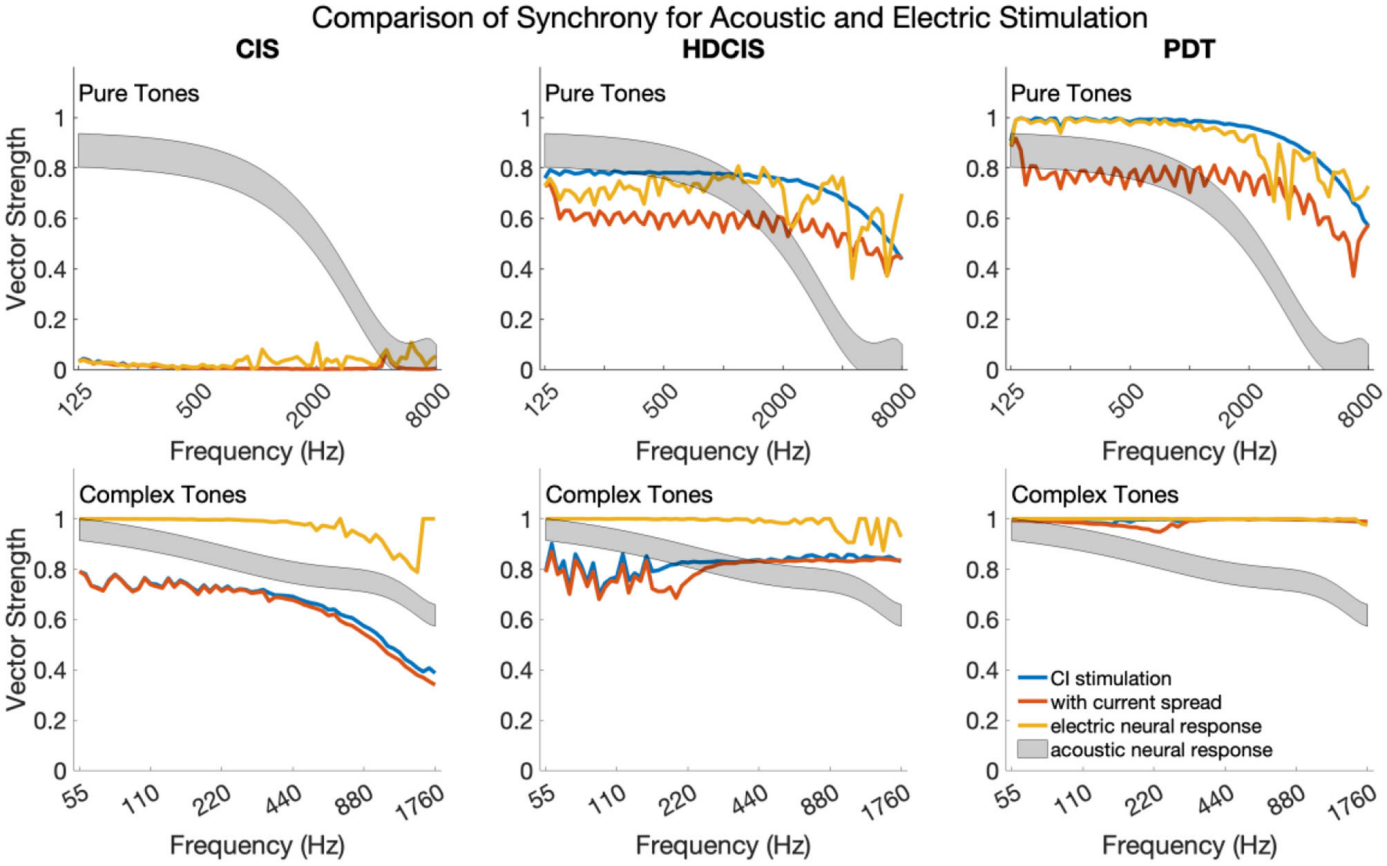
Synchrony of neural response to the frequency of pure tones and fundamental frequency of complex tones for acoustic and electric stimulation.

Figure 6 shows synchrony quantified as vector strength for modeled auditory nerve fibers for acoustic and electric stimulation. The upper and lower subpanels show synchrony in response to pure and complex tones, respectively. In each subpanel, the acoustic neural response is the vector strength based on the phenomenological auditory-nerve model. The different columns of subpanels show the synchrony of the electric stimulus before and after current spread and neural modeling with the point process model.

For pure tones, modeled acoustic neural response captures the diminishing of neural synchrony that occurs in normal hearing for frequencies above 2–4 kHz. The CIS stimulation strategy simply does not convey temporal fine structure, which is a known design flaw. The HDCIS and PDT strategies both provide a high level of synchrony in the stimulation pattern, which is degraded by current spread, but mostly restored by the point process model of neural excitation. Neural synchrony does not diminish near 2 kHz for electrical stimulation, which agrees with physiological data suggesting that neural synchrony is higher for electric than for acoustic stimulation since electric stimulation bypasses the sluggish synaptic mechanisms of transduction (Dynes and Delgutte, 1992). The vector strength of response for electric stimulation does diminish above 4 kHz, which is driven by the electric pulses not being perfect impulses but having finite durations (*i.e*., the total pulse width of about 60 μs is a significant portion of the tonal period). The most relevant finding is that the synchrony of modeled neural response to pure tones is comparably high for HDCIS and PDT when compared to synchrony observed in acoustic hearing. The observed synchrony in the modeled neural response is consistently higher than that observed in modeled acoustic hearing for the PDT stimulation strategy.

The modeled neural response for acoustic stimulation is similar for complex tones as for pure tones. As depicted in Figure 1, the vector strength for complex tones has two distinct regions with high levels of synchrony: one associated with the fundamental component and one associated with the high-frequency spectral regions of unresolved harmonics. The maximum vector strength across fibers is shown for complex tones as a summary statistic. For the model of auditory-nerve response to acoustic stimulation, synchrony to the fundamental follows the same behavior as a pure tone. Synchrony is relatively high for the three stimulation strategies. For CIS, since synchrony is not provided for the fundamental, the observed synchrony derives from the high-frequency spectral region of unresolved harmonics. As can be seen in Figure 3, the temporal periodicity of stimulation in high-frequency channels are in phase and thus are not degraded by current spread. Modeled neural excitation increases vector strength since the point process neurons tend to respond during the same phase of the charge accumulation. Observed synchrony was higher yet for HDCIS and PDT with both being enhanced by modeled neural response. While parameterization of the computational model will affect results, neural excitation to electric stimulation clearly provides temporal cues with comparable neural synchrony in acoustic hearing.

### Effect of Stimulation Rate on Neural Response

**Figure 7:**
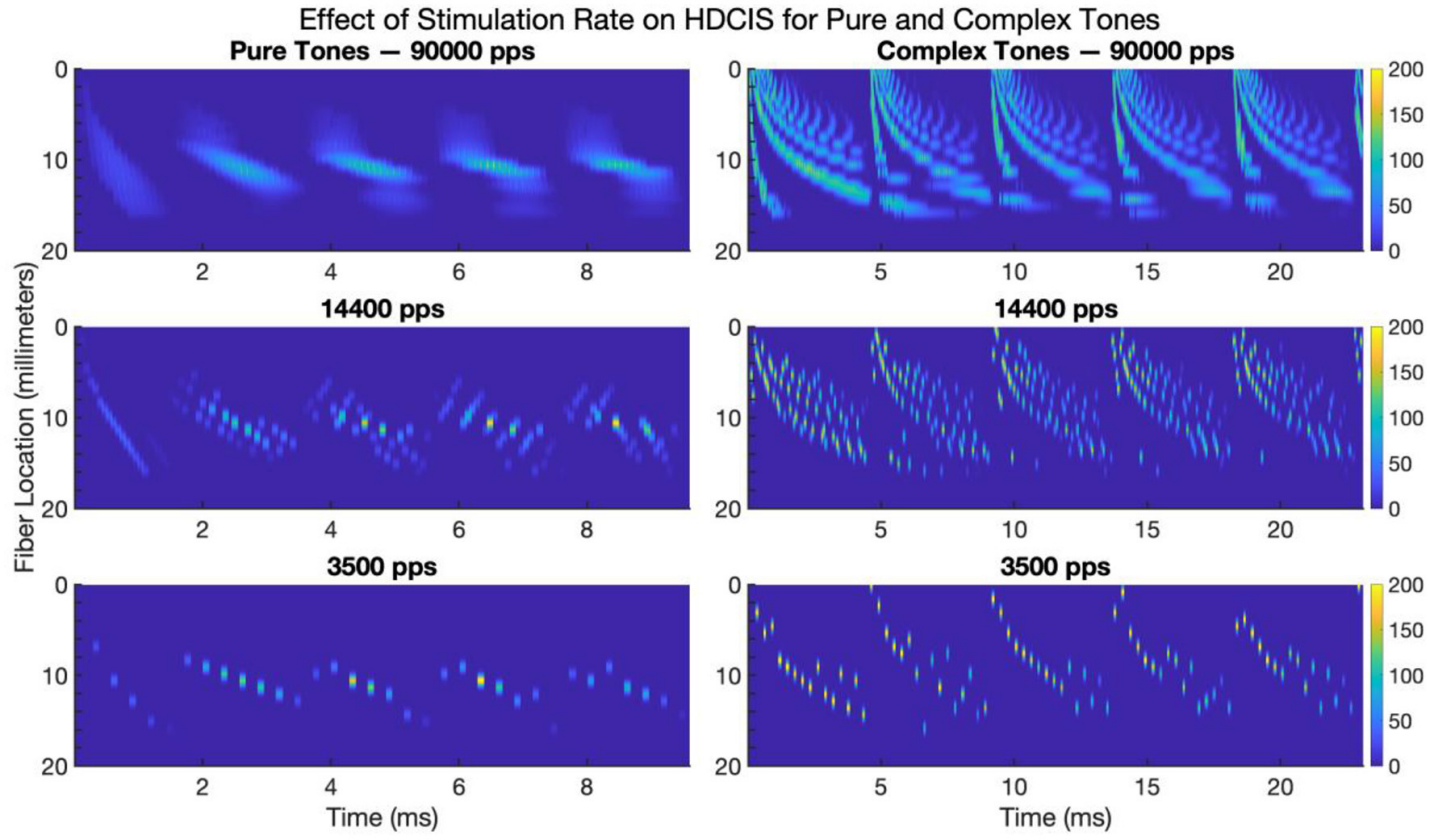
Modeled neural response to electrode stimulation for pure and complex tones for high-definition emulations of the Advanced Combinatorial Encoders stimulation strategy. The images show modeled fiber location vs. time for five cycles of a 500 Hz pure tone (left panels) and five cycles of a 220 Hz complex tone (right panels). The color bar indicates the average firing rate of the neural response. pps, pulses per second.

Figure 7 shows the modeled neural response for HD-ACE implemented for the three stimulation rates for pure and complex tones. Only the first 20 millimeters of the fiber locations are shown since the modeled 22-electrode array only extends to be perpendicular to the 16-millimeter location. The cycle-by-cycle temporal fine structure for both pure and complex tones are conveyed by the stimulation strategy but with noticeable reduction in the amount of fine-structure detail that is encoded.

These modeling results clarify the effect of stimulation rate on encoding temporal fine structure into neural activity. While some degree of synchrony is maintained despite the reduction in stimulation rate, there is clearly a loss of detail in the response of the auditory nerve. Psychophysically, a previous study has shown that cochlear implant users are sensitive to small fluctuations in charge density that occur associated with this loss of detail in the temporal fine structure (Goldsworthy et al., 2022).

## Discussion

Neural synchrony to incoming stimulation is exceptionally high in the auditory system. The auditory nerve fires with synchrony to incoming sounds for frequencies up to two and arguably as high as 10 thousand cycles per second (Dynes and Delgutte, 1992; Chung et al., 2014, 2019; Verschooten et al., 2019). This remarkable synchrony was examined here by comparing computational models of auditory-nerve response to acoustic and electric stimulation. It was shown that synchrony to sound of the most common stimulation strategies for cochlear implants (ACE and CIS) is completely discarded for pure tones and substantially degraded for complex tones, specifically that the temporal fine structure of the fundamental frequency is discarded. In contrast, modeling results indicate that stimulation strategies that explicitly encode temporal fine structure (HDCIS and PDT) provide comparable levels of neural synchrony to sound as produced in acoustic hearing. Modeled current spread reduced spatial and temporal cues for pitch perception, but the point process model of neuronal response increased both spatial specificity and neural synchrony. Discussion focuses on how computational modeling could be used to yield better encoding of temporal cues for cochlear implants.

The present article compares modeled neural synchrony for different cochlear implant stimulation strategies with synchrony observed in acoustic hearing. The ACE and CIS strategies are commonly used with cochlear implants, but it is well-known that these strategies discard temporal fine structure of sound. The extent, however, that computational modeling indicates that HDCIS and PDT encode temporal fine structure of incoming sound into synchronous neural activity is enticing. Of course, aspects of modeling such as the extent of current spread and the distribution of neural parameters will affect modeling results, and this flexible capacity will allow models to be tuned to physiological data collected in the future. Since HDCIS encodes aspects of temporal fine structure into stimulation, and does not explicitly remove that information using smoothed envelopes, some extent of neural synchrony will be transmitted. Similarly, stimulation strategies that trigger stimulation pulses based on temporal fine structure such as PDT, FSP, and FS4 will produce varying degrees of neural synchrony (van Hoesel, 2007; Vandali and van Hoesel, 2011; Riss et al., 2014, 2016). The present article does not attempt to exhaustively describe synchrony for all strategies, but to clearly describe synchrony for common stimulation strategies.

The modeling results presented here incorporate current spread and a point process model or neuronal response. Synchrony was degraded by current spread because the front-end filtering included phase delay like that produced by traveling wave mechanics of the cochlea. If linear phase filtering were instead used for the front-end filtering—as often used for cochlear implant signal processing—then stimulation timing features would be coherent and current spread would cause less smearing of temporal information. However, it is not presently known the extent that modeling traveling wave mechanics is important for conveying temporal fine structure cues (Loeb et al., 1983; Loeb, 2005; McGinley et al., 2012). Computational modeling can be used to clarify the tradeoffs between emulating traveling wave mechanics and minimizing the smearing of temporal cues for pitch perception (Cohen, 2009; Karg et al., 2013; Seeber and Bruce, 2016; van Gendt et al., 2016).

There is a rich body of literature associated with temporal cues for pitch perception in the auditory-nerve response with strong arguments for different metrics to quantify the strength of these cues (Meddis and Hewitt, 1992; Cariani and Delgutte, 1996a,b; Cedolin and Delgutte, 2010; Hartmann et al., 2019). Vector strength was made the focal point of the present article as a starting point for comparing the temporal response properties of the auditory nerve to acoustic and electric stimulation because it is a straightforward metric of synchrony that has been used in basic studies of physiology (van Hemmen, 2013). Further, interval histograms were examined and the periodicity information present in the neural response to electrical stimulation was comparable to that observed for acoustic stimulation (Mckinney and Delgutte, 1999). Future work should consider other metrics of synchrony as predictors of behavioral pitch resolution as results are better characterized for different stimulation strategies.

The extent that encoding synchrony into sound processing for cochlear implants will improve music and speech perception for recipients is unknown. The results of the present study show that synchrony is poorly encoded by conventional ACE and CIS strategies, but that synchrony can be restored using stimulation based on physiology that actively encodes the temporal fine structure of incoming sound. Behavioral results for strategies that attempt to encode temporal fine structure (*e.g*., HDCIS, FSP, PDT) have yielded promising, but mixed results (van Hoesel and Tyler, 2003; van Hoesel, 2007; Vandali and van Hoesel, 2011). The most promising results for such strategies suggest that prolonged rehabilitation is needed to make use of newly encoded timing cues, which suggests long-term recovery and rehabilitation of neural circuits tuned to synchronous activity (Kral and Tillein, 2006; Kral and Lenarz, 2015; Riss et al., 2016). Musical sound quality was shown to be better with FSP compared to HDCIS particularly for bass frequency perception (Roy et al., 2015). New strategies designed to encode temporal fine structure should be evaluated with prolonged periods of experience. Further, optimization of new strategies could benefit by modeling how synchrony to sound can be restored first at the level of the stimulation pattern but ultimately at the level of the auditory nerve.

## Data Availability Statement

The modeling code and results supporting the conclusions of this article will be made available by the authors, without undue reservation.

## Author Contributions

The author confirms being the sole contributor of this work and has approved it for publication.

## Funding

This research was supported by a grant from the National Institute on Deafness and Other Communication Disorders (NIDCD) of the National Institutes of Health: R01 DC018701. The funding organization had no role in the design and conduct of the study; in the collection, analysis, and interpretation of the data; or in the decision to submit the article for publication; or in the preparation, review, or approval of the article.

## Conflict of Interest

The author declares that the research was conducted in the absence of any commercial or financial relationships that could be construed as a potential conflict of interest.

## Publisher's Note

All claims expressed in this article are solely those of the authors and do not necessarily represent those of their affiliated organizations, or those of the publisher, the editors and the reviewers. Any product that may be evaluated in this article, or claim that may be made by its manufacturer, is not guaranteed or endorsed by the publisher.
